# Robotic-assisted patellofemoral arthroplasty provides excellent implant survivorship and high patient satisfaction at mid-term follow-up

**DOI:** 10.1007/s00264-024-06224-2

**Published:** 2024-05-31

**Authors:** Giacomo Pacchiarotti, Alessandro Todesca, Michele Coppola, Stefano Gumina

**Affiliations:** 1 Department of Anatomy, Histology, Legal Medicine, and Orthopaedics, Sapienza University of Rome, Rome, Italy; 2 Istituto Chirurgico Ortopedico Traumatologico (ICOT), Latina, Italy

**Keywords:** Partial knee arthroplasty, Unicompartimental Arthroplasty, Patellofemoral Arthroplasty, Patellofemoral Osteoarthritis, Robotic-assisted surgery

## Abstract

**Purpose:**

Robotic adoption in knee surgery has yielded several benefits, but its application in patellofemoral arthroplasty (PFA) remains barely reported. The purpose of this study was to determine implant survival, patient satisfaction, and functional outcomes after robotic-assisted PFA at an intermediate follow-up.

**Methods:**

This prospective analysis targeted 18 knees of 16 consecutive patients who underwent robot-aided PFA with three-year minimum follow-up (range, 3 to 6 years). Each patient was evaluated collecting pre-operative and post-operative medical record data, including range of motion, radiographic images, and multiple scores, such as VAS, APKS, and OKS.

**Results:**

At surgery, the mean age was 55.4 years ± 14.4 (range, 32 to 78 years), and the mean BMI was 26.8 kg/m² ±5.2 (range, 20 to 36). Etiologies of patellofemoral osteoarthritis included idiopathic degeneration (28%), post-traumatic (33%), and dysplasia (39%). Pre-implantation scores were VAS 7.9 ± 1.4, AKPS 34.6 ± 23.3, and OKS 17.3 ± 10.3. One implant was revised with primary total knee arthroplasty for osteoarthritis progression. Clinical and radiographic follow-up showed no signs of loosening or infection. The maximum flexion reached an average of 131.1°±10.5° (range, 110° to 145°), accompanied by significantly improved score results (*P*-value < 0.01): VAS 1.1 ± 1.4, AKPS 90.2 ± 8.6, and OKS 46.3 ± 1.8.

**Conclusions:**

At 3 years after robotic assisted patellofemoral arthroplasty, excellent implant survival and patient satisfaction rates can be expected along with significantly improved functional and pain control outcomes. Although the limitations imposed by the restricted cohort, these findings indicate that robotic assistance in PFA is both safe and effective at intermediate follow-up.

## Introduction

Patellofemoral joint osteoarthritis (PFOA) affects 40% of individuals with knee pain, appearing radiographically isolated in approximately 10% of the middle-elderly aged population [1–3]. Among the possible treatments, patellofemoral arthroplasty (PFA) emerges as a viable option, addressing degenerated bone surfaces, correcting trochlear deformities, and enhancing patellofemoral tracking [4]. Regardless the epidemiological trend, PFA constitutes merely 1% of knee replacements, while total knee arthroplasty (TKA) remains the most widely implanted option [5, 6]. This happens despite TKA ensures inferior outcomes compared to single-compartment replacement in terms of bleeding, operative time, hospital stay, and post-operative recovery [7–11]. Moreover, prosthesizing two healthy compartments in young and active patients can lead, over time, to more numerous and invasive procedures, while PFA can be converted to TKA with good outcomes [12–14]. Compared to TKA, PFA preserves bone stock and further, does not involve femoro-tibial interface, neither causes alignment issues throughout the range of motion.

However, these implants have been burdened over time by a high failure rate primarily linked to osteoarthritis (OA) progression and femoral component malpositioning [15]. Poor prosthesis placement has been associated with increased failure and a higher revision rate observed with PFA in comparison with TKA. Surgeons undertaking low volumes of PFA have higher revision rates, reflecting the complexity of the surgery [14, 16].

In this landscape, accuracy and precision offered by robotic surgery stands out as a valuable source of assistance. This technology combines the advantages of virtual planning, introduced via navigation techniques, with high accuracy in bone shaping, significantly enhancing precision and reproducibility in implant positioning [17–20]. Strong evidence demonstrates its results in TKA and femoro-tibial unicompartimental knee arthroplasty (UKA) improving implant sizing, limb alignment, and soft tissue balancing [21–23]. This innovation has the potential to impact the efficacy of PFA, reducing failure rates and theoretically extending its survival [24]. Despite this technology has become an attractive method to ensure an accurate execution of the surgical plan, its integration in PFAs remains barely reported and further, its outcomes are confined to the short-term.

The purpose of this study was to determine implant survivorship, complications, re-operation rates, and further investigate functional and pain control outcomes and overall satisfaction after robotic-assisted PFA at intermediate follow-up.

## Methods and materials

### Study population

This prospective study encompassed 18 consecutive patients (20 knees) who underwent anterior knee compartment replacement with PFA spanning from January 2018 to January 2021. This cohort constituted the initial series for the Journey® Patellofemoral Joint System (Smith & Nephew Inc., Andover, UK) placed in a single centre, with prior experience in navigated and robot-aided implant placement, with NAVIO® imageless robotic-assisted surgical system (BlueBelt Navio Robotic Surgical System - Smith & Nephew®).

Due to the rarity of the procedure, inclusion criteria enclosed all primary causes of PFOA, comprising trochlear dysplasia, idiopathic osteoarthritis, and OA resulting from trauma. Within our cohort, every trauma induced PFOA was generated from patellar fractures. All participants exhibited radiographic evidence of severe isolated patellofemoral joint OA (narrowing of the joint space, subchondral sclerosis, and cyst formation) associated with anterior knee pain. Each patient had symptoms persisting for over a year, intensified by activities stressing the patellofemoral joint, impeding work and daily tasks, and unresponsive to conservative treatments [25, 26]. Individuals with inflammatory knee arthropathy, femoro-tibial instability, tibiofemoral OA (Kellgren-Lawrence II or greater), femoro-tibial lesions > 6 mm in diameter, low patella, varus or valgus deformity > 5°, and decreased range of motion (minimum ROM − 10° of extension and 110° of flexion) were excluded. [25, 27, 28].

### Surgical technique

**Fig. 1:**
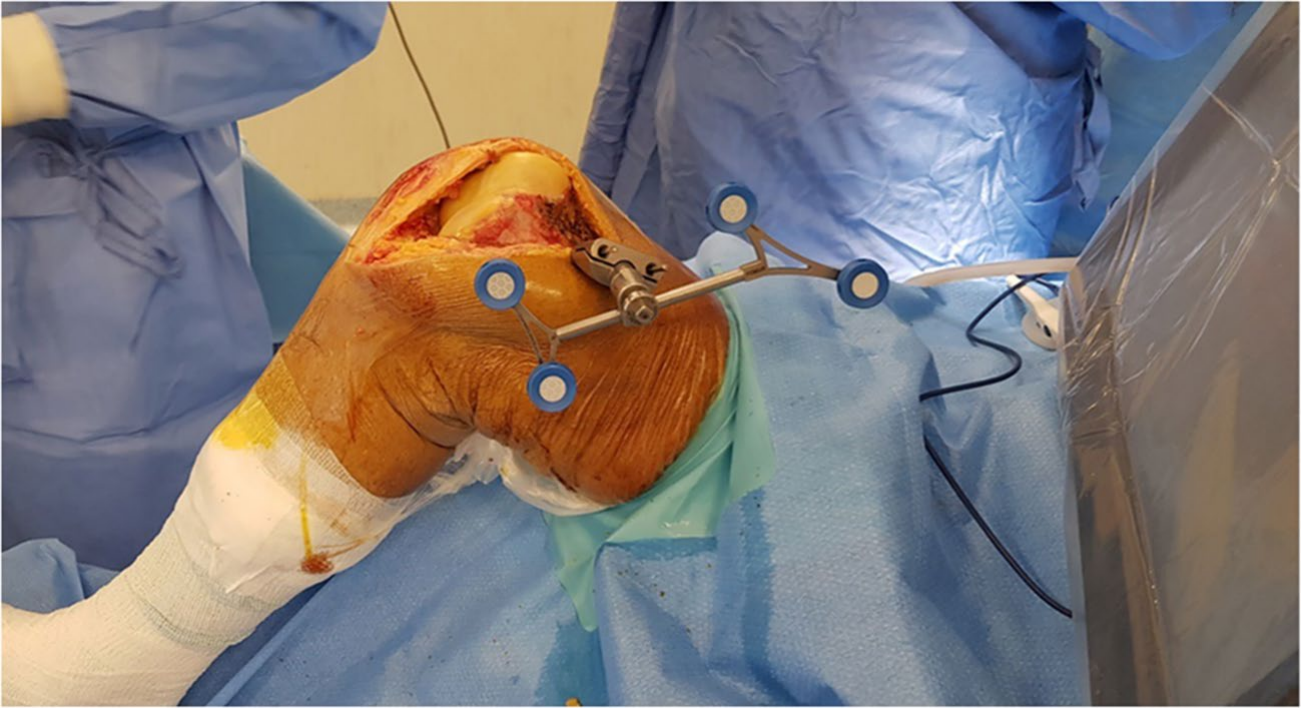
Femoral tracker placement within the surgical wound using two meta-diaphyseal arrays

**Fig. 2:**
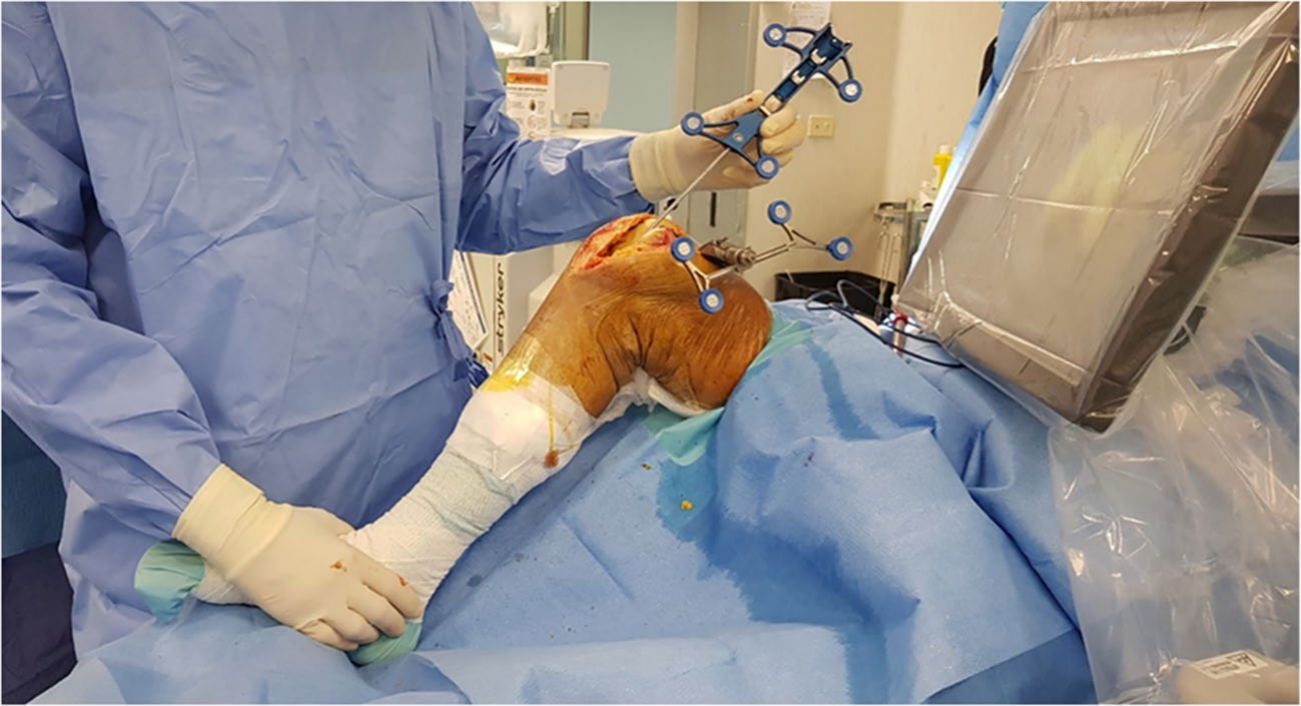
Acquisition of the first anatomical landmark: the Whiteside line

**Fig. 3:**
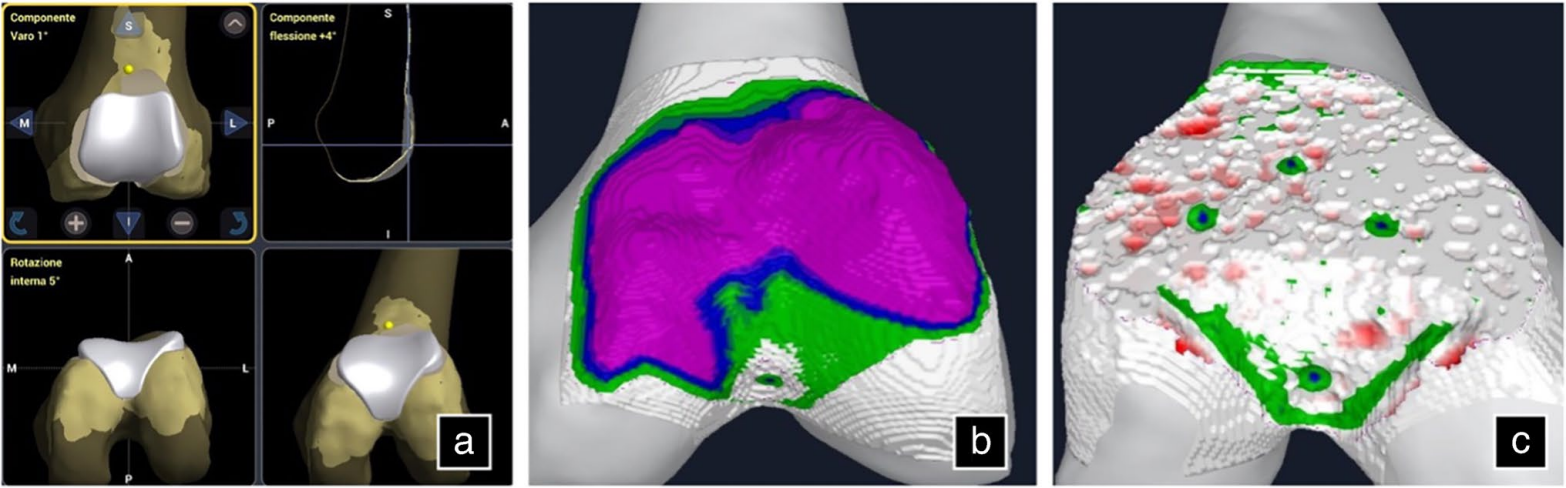
Imageless systems produce a virtual 3D reconstruction of the patient’s distal femoral epiphysis allowing precise implant positioning and ensuring its accurate execution. (**a**) Planning of component sizing and orientation on every plane. (**b**) Digital representation of the bone surface before burring. The bony prominences to remove are represented using a chromatic scale. (**c**) Bone surface after burring. Upon reaching the target bone surface (white) the drill retracts actively

**Fig. 4:**
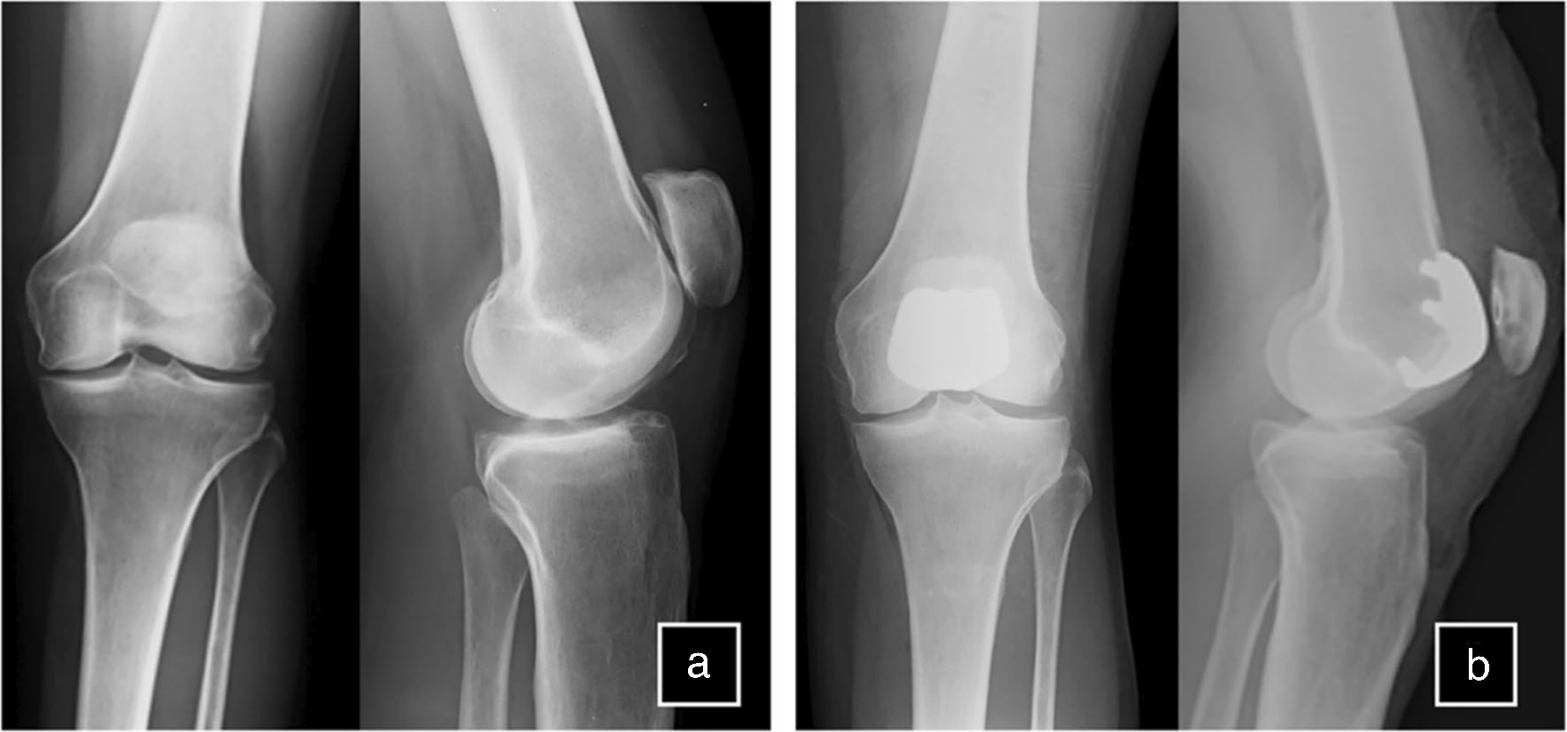
Patellofemoral arthroplasty implantation with imageless robotic-assisted surgical system. Anteroposterior and lateral radiograph of a 61-year-old patient with isolated patellofemoral osteoarthritis (**a**) before surgery and (**b**) after surgery

The surgical procedures were performed under spinal anaesthesia through a midvastus approach. Lateral eversion of the patella facilitated the examination of the entire distal femoral epiphysis, assessing cartilage integrity (absence of chondral lesions or arthritic degeneration) in both femoro-tibial compartments. Osteophytes were removed if present. A tracker was positioned on the femoral metaphysis using two arrays within the surgical wound (Fig. 1). The first, located approximately a hand span (four fingers) superior to the patella, faced to the center of the femur [29], while the second was fixed parallel to the first through a dedicated guide. Virtual model of the patient’s distal femur and trochlea was generated intraoperatively digitizing anatomical landmarks with an infrared camera-guided point probe without any pre-operative MRI or CT imaging. The surgeon first identified femoral reference points, including the Whiteside line (Fig. 2), the centre of the knee, the femoral mechanical axis, the expected termination point of the implant on the anterior femoral cortex, and the trans-epicondylar axis (TEA) through the recognition of the most prominent points over the medial and lateral epicondyles. Then, proceeded to the “bone morphing” moving the probe over the anterior femoral cortex and trochlear sulcus surface. The system provided a virtual reconstruction of the patient’s cartilage and bone morphology, enabling visualization of the implant’s position in axial, sagittal, and coronal planes, with a 3D representation. Correct sizing was established with the medio-lateral native limits of the condyles. Coronal and sagittal alignments were determined considering the femoral mechanical axis, the trochlear proximal limit, and the most anterior and antero-distal points of the trochlear sulcus to ensure either a smooth engagement and a correct tracking without overstuffing. Axial alignment was established using the Witheside line, with an additional correction of 1 to 5 degrees of internal rotation, to address any pre-existing deformities (Fig. 3a). Afterwards, the femur was shaped with a high-speed intelligent bur. This burring system remains extended and active only until the target bone surface is reached, and its exposure is actively adjusted to minimize the risk of overcut. The monitor displayed a chromatic scale, guiding the surgeon until the target surface (white) was reached (Fig. 3b and c). The patella was prepared with a conventional ancillary technique. Trial components were positioned, and the arthroplasty was tested along the entire range of motion confirming patellar tracking and stability. Finally, the femoral metal and dome-shaped all-poly patellar components were cemented (Fig. 4).

### Data collection

Before surgery, each patient was informed that his operation would have been robotically assisted and underwent functional and pain assessment with multiple questionnaires including Visual Analogue Scale (VAS), Kujala Anterior Knee Pain Scale (AKPS), Oxford Knee Score (OKS). Radiographic measurements of hip-knee-ankle (HKA) angle, Caton-Deschamps Index [30], and Dejour trochlear Classification [31] were also collected. In January 2024, with a minimum follow-up of three years (3–6 years), all patients were visited collecting medical record, physical examination, and recent knee X-Rays. Overall satisfaction was assessed on a 5-level Likert scale: “high dissatisfaction”, “dissatisfaction”, “neutral”, “satisfaction”, or “high satisfaction”. Functional and pain control outcomes were evaluated administering the same questionnaires to each patient. Any adverse event or complication, and its etiology at any timepoint was recorded. Each patient was contacted multiple times before being considered lost.

#### Data analyses

Collected data were tabulated in a Microsoft Excel ® sheet (Microsoft Corporation, Redmond, Washington, USA) and analyzed using SPSS software, version 22.0 (IBM-SPSS, New York, USA). Descriptive statistical analysis was reported with mean, median, range and standard deviation values with 95% confidence interval. The nonparametric Wilcoxon test for two related samples was used to compare pre-operative and post-operative results of each function score. All statistical tests were performed two-sided. Statistical significance was considered at *P*-value < 0.01 for all analyses. There were insufficient data to perform survival analysis with the Kaplan-Meier method.

## Results

Among the 18 patients who underwent robotic-assisted PFA surgery, two were lost to follow-up (could not be contacted). The study encompassed a cohort of 16 patients, consisting of nine men and seven women. The distribution involved eight rights, six left, and two bilateral cases, totaling 18 knees. At surgery, the mean age was 55.4 years ± 14.4 (range, 32 to 78 years), and the mean BMI was 26.8 kg/m² ±5.2 (range, 20 to 36). The etiology of PFOA included idiopathic degeneration in 28%, post-traumatic causes in 33%, and trochlear dysplasia in 39% of cases. Seven knees had evidence of trochlear dysplasia (eleven type A, two type B, four type C, and one type D). Demographic data, radiographic findings and pre-surgery knee history and are detailed in Table 1.

**Table 1:** Patient demographics and peri-operative data, including radiographic findings, implant survival and overall satisfaction at the last follow-up

Age at surgery (years old)	55.4 ± 14.4
Body mass index (kg/m^2^)	26.8 ± 5.2
Gender distribution (*n*)	
Male	9
Female	7
Side (%)	
Left	50 (*n* = 8)
Right	37.5 (*n* = 6)
Bilateral	12.5 (*n* = 2)
Etiology (%)	
Idiopathic	28 (*n* = 5)
Post-traumatic	33 (*n* = 6)
Dysplasia	39 (*n* = 7)
Follow-up (months)	52 ± 12.6
Blood Loss (ml)	2.12 ± 0.6
Length of surgery (min)	81 ± 22
Length of hospital stay (days)	4.6
HKA angle (°)	176.7 ± 1.5
Caton-Deschamps Index	1.0 ± 0.2
Dejour Classification (%)	
A	61 (*n* = 11)
B	11 (*n* = 2)
C	22 (*n* = 4)
D	6 (*n* = 1)
Reoperation/ implant survival (%)	94%
Overall satisfaction (%)	
High satisfaction	61 (*n* = 11)
Satisfaction	33 (*n* = 6)
Dissatisfaction	6 (*n* = 1)
Maximum Flexion (°)	131.11 ± 10.5
Maximum Extension (°)	1.67 ± 3.5

Parametric data expressed as mean values ± standard deviation. HKA angle, Hip-Knee-Ankle angle

Pre-implantation symptoms were documented with multiple scores: VAS 7.9 ± 1.4 (range, 4.9 to 10), AKPS 34.6 ± 23.3 (range, 7 to 85), and OKS 17.3 ± 10.3 (range, 10 to 41). The average operating time was 81 min ± 22 (range, 60 to 120), the average hospital stay was 4,6 days (range, 3 to 6), while bleeding, calculated as the difference between pre-operative and second post-operative day blood count, was Hb 2.12 ± 0.6 (range, 1.2 to 3.4) without blood transfusions. No patient underwent further surgical gestures to center the patella (e.g. lateral release, tibial tuberosity transposition, MPFL reconstruction).

The average time elapsed from surgery to the latest follow-up was 52 months ± 12.6 (range, 36 to 72). The implant survivorship was high (94.4%) with no surgical or robot-technique related complication recorded. Only one patient experienced, after five years, OA progression in the femoro-tibial compartment, requiring primary total knee arthroplasty revision. Every patient expressed satisfaction or high satisfaction with noticeable enhancement in their quality of life, except for one patient with high pre-operative functional scores (OKS 41), who maintained comparable post-operative results, not reaching his expectation. Radiographic control, performed close to the visit, showed no signs of implant loosening or further OA progression. At the last follow-up, final ROM was excellent in each patient, with mean maximum flexion of 131.1°±10.5° (range, 110° to 145°), while functional scores were VAS 1.10 ± 1.4 (range, 0 to 4), AKPS 90.2 ± 8.6 (range, 73 to 98), and OKS 46.3 ± 1.8 (range, 43 to 48). Pain and functional scales demonstrated significant outcome improvement (*P*-value < 0.01) between pre-operative and post-operative assessments (Tables 2 and 3).

**Table 2:** Comparison of pre-operative and post-operative functional and pain control outcomes

	Preoperative		Postoperative		
	Mean ± SD	(Range)	Mean ± SD	(Range)	*P-*values*
VAS	7.99 ± 1.4	(4.90 to 10.00)	1.10 ± 1.4	(0.00 to 4.00)	< 0.01
AKPS	34.56 ± 23.3	(7.00 to 85.00)	90.2 ± 8.6	(73.00 to 98.00)	< 0.01
OKS	17.3 ± 10.3	(10.00 to 41.00)	46.3 ± 1.8	(43.00 to 48.00)	< 0.01

SD, standard deviation; VAS, Visual Analogue Scale; AKPS, Kujala Anterior Knee Pain Scale; OKS, Oxford Knee Score. *Differences between pre-operative and post-operative values (Wilcoxon test)

**Table 3:**
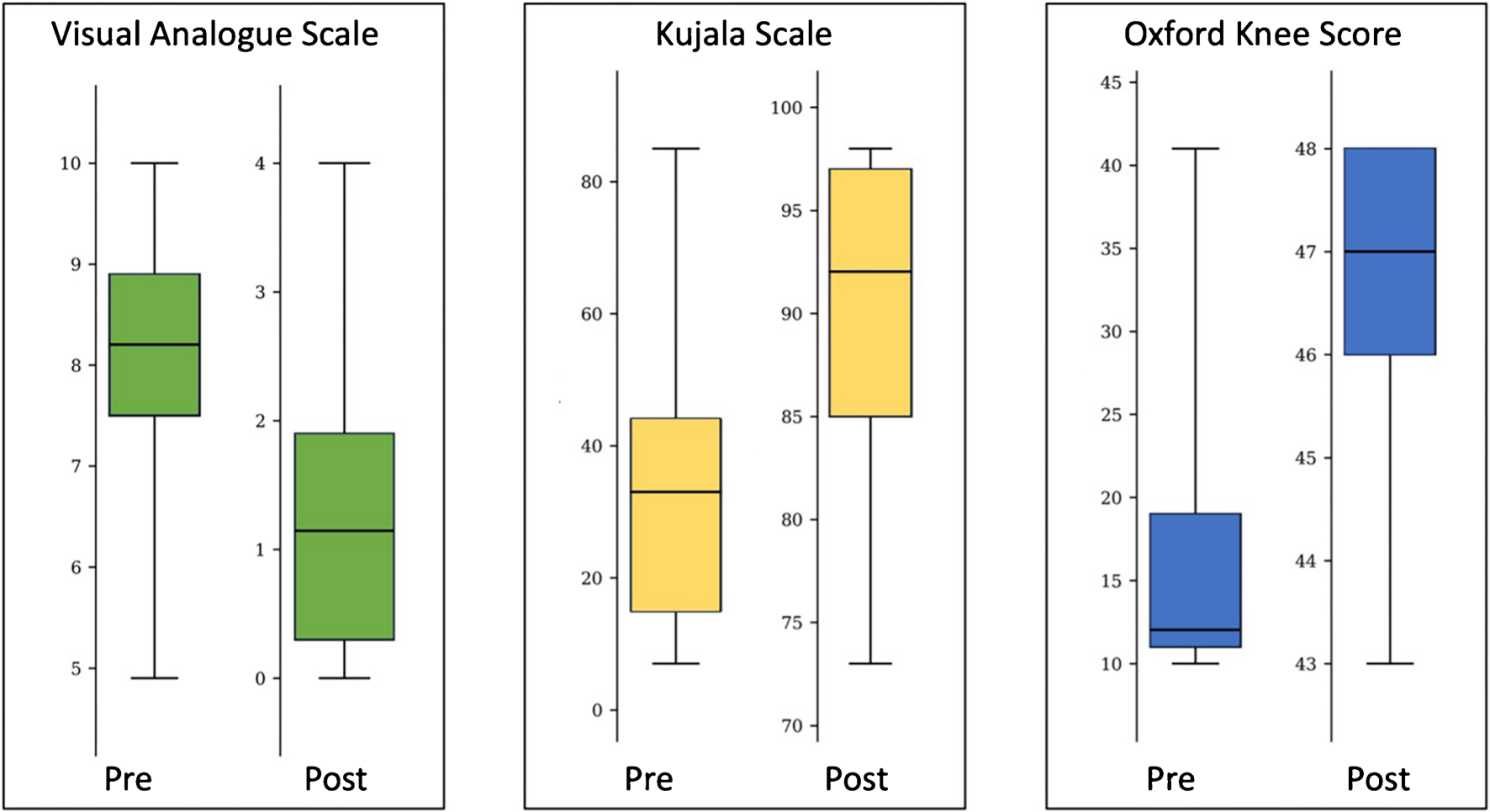
Visual representation of pre-operative and post-operative functional and pain control outcomes

## Discussion

This is the first series of robotic-assisted PFAs assessed at a minimum follow-up of three years after surgery. We have reported high overall satisfaction and implant survival rates. Almost every patient resolved their primary symptoms raising their quality of life. Administered questionnaires recorded significant enhancement in functional and pain control outcomes at a mean follow-up of four years: VAS improved from 7.9 to 1.10, APKS from 34.6 to 90.2, and OKS from 17.3 to 46.3. We attribute these outcomes to a combination of meticulous patient selection criteria and proper surgical procedure.

Robotic technology plays a key role simplifying the procedure and reducing PFAs positioning outliers. As these implants need to integrate with native kinematics, they are affected by minimal spatial incongruities which can lead to instability, subluxations, catching, snapping, patellar maltracking, as well as anterior knee pain [32–34]. Furthermore, positioning errors may produce an improper force distribution over the implant, leading to adverse effects such as excessive wear of the patellar all-polyethylene component and aseptic loosening in both short and long-term [9, 32, 34–36]. Malposition emerges than as the main cause for implant revision along with OA progression [14, 37].

Enhanced implant positioning and greater reproducibility have been, over time, the primary objective behind every development and advancement in PFA history [38]. First–generation “inlay” prostheses were small and narrow. These implants exhibited elevated rates of maltracking and failure arising from positioning errors as result of freehand cutting technique. Moreover, as inlay PFAs positioning relied on the native femoral epiphysis, in the context of high-grade trochlear dysplasia, achieving optimal rotation and implant positioning on the femur might be very challenging even for seasoned experts [15, 39]. Second-generation “onlay” prosthesis addressed positioning errors introducing a reproducible technique based on a TKA-fashion anterior knee cut. This advancement mitigates trochlear internal rotation risk and effectively manages any degree of dysplasia demonstrating a tangible impact on reducing failure rates [16, 38, 39]. However, these implants can’t effectively substitute the native trochlea, accelerating the progression of OA, which persist as great cause of failure [40–42]. Moreover, 2nd generation PFAs often result in an increased thickness of the trochlea, causing overstuffing of the patellofemoral joint and, consequently, pain due to increased tension exerted on the retinacular fibers [43]. Additionally, a slight flexion or extension of the intramedullary guide can easily lead to flexion or extension of the trochlear component, resulting in patellar catching [39, 44, 45].

Robotic systems can address these challenges creating a computer-aided design model of the patient’s knee joint, guiding a precise bone resection, and positioning the implant within an error of 1 mm [18, 19]. Diverse technologies are employed: Image-based systems utilize pre-operative planning based on CT models validated during the procedure, while imageless systems rely on infrared cameras and point probes for three-dimensional femoral anatomy reconstruction and a subsequent full intraoperative planning. This technology could potentially combine the benefits of both generations, enabling an extremely precise, reproducible, and anatomical-integrated positioning on the femoral trochlea. Imageless systems are particularly suitable for partial knee replacement as they rely on the cartilage surface rather than bony margins, accurately positioning the implant embedded within the cartilage.

Navigated and robot-aided surgery demonstrated significantly enhanced lower limb alignment, soft tissue balance, and in implant positioning [17, 20]. Particularly, in tibio-femoral unicompartimental replacements has yielded significant advantages reducing complication and revision rates, heightening prosthetic positioning accuracy with fewer outliers, and enhancing range of motion and PROMs [21–23]. The robotic adoption in PFAs procedures serves a dual purpose: firstly, it seeks to replicate the precision and accuracy observed in UKA and TKA surgery and, secondly, it aims to facilitate the procedure’s adoption in lower-volume centers due to the high reproducibility and the short learning curve [46, 47]. Currently, PFA is a niche procedure performed almost exclusively in high-volume centers [14]. Batailer et al., in the sole comparative study reports comparable short-term functional outcomes and failure rates between robotic-assisted and conventional PFAs performed by skilled operators in high-volume centers [48]. Robotic systems can provide a crucial contribution to ensure an accurate planning and its executions, encouraging less experienced surgeons to embrace the procedure [49].

In our experience, intraoperative planning is crucial for achieving patellar centering across the range of motion and preventing misplacement errors. Both intrarotation and overstuffing can be avoided establishing desired rotation and depth respect to the trochlear surface and the many reference axes on the virtual model before the actual bone burr [48]. Implant misplacements along the sagittal plane such as hyperflexion or hyperextension and sizing errors like overstuffing or overhanging on the femoral condyles can be easily prevented as well [32]. In this series, no additional surgical patellar centering procedures, or lateral releases, often necessary in conventional PFA, were required in any patient [26, 34]. Finally, patients undergoing partial knee arthroplasty are usually young with high expectations of returning to their previous activity level, especially in sports [50].

The PFA cohort presented in this study constitutes 8% of imageless robot-assisted procedures and around 2.5% of the total knee replacement volume performed by our group in the same timeframe. This data highlights our trust in this technology to impact the efficacy of PFA. We believe that achieving a reproducible implant positioning and a good integration with physiological knee kinematics could ensure long-term survival.

This study has several limitations that should be noted, including the limited number of patients, and the absence of a direct comparison between robotic-assisted and conventional PFA, precluding any conclusions on the superiority of either approach. Moreover, outcomes assessment may be influenced by the diverse PFOA etiologies and patient awareness of the robotic-system adoption. Further, they were the firsts to receive a PFA positioned with robotic assistance, representing a single-surgeon (TA) case series.

## Conclusion

At three years after robotic assisted patellofemoral arthroplasty, excellent implant survival and patient satisfaction rates can be expected along with significantly improved functional and pain control outcomes. The sole reported failure is attributed to tibio-femoral osteoarthritis progression. Although the limitations imposed by the restricted cohort, these results indicate that robotic assistance in PFA is both safe and effective at intermediate follow-up representing an incentive for a wider study of robotic systems in patellofemoral replacement.

## Declarations

All procedures performed in studies involving human participants were in accordance with the ethical standards of the institutional and/or national research committee and with the 1964 Helsinki declaration and its later amendments or comparable ethical standards.
